# Development of a food preservative from sea buckthorn together with chitosan: Application in and characterization of fresh-cut lettuce storage

**DOI:** 10.3389/fmicb.2023.1080365

**Published:** 2023-03-07

**Authors:** Kexin Feng, Xiaolin Feng, Weijian Tan, Qinhua Zheng, Wenting Zhong, Caiyu Liao, Yuntong Liu, Shangjian Li, Wenzhong Hu

**Affiliations:** ^1^College of Pharmacy and Food Science, Zhuhai College of Science and Technology, Zhuhai, China; ^2^College of Life Science, Jilin University, Changchun, China

**Keywords:** flavonoid, sea buckthorn leaf, chitosan, compound preservative, fresh-cut lettuce

## Abstract

The purpose was to create a novel composite food preservative for fresh-cut lettuce using flavonoids and chitosan from sea buckthorn leaves (SBL). Sea buckthorn leaves were extracted with ethanol as the extraction solvent and ultrasonic-assisted extraction to obtain flavonoid from sea buckthorn leaf crude (FSL), and then the FSL was secondarily purified with AB-8 resin and polyamide resin to obtain flavonoid from sea buckthorn leaf purified (FSL-1). Different concentrations of FSL-1 and chitosan were made into a composite preservative (FCCP) by magnetic stirring and other methods, containing 1% chitosan preservative (CP) alone, 0.5–2 mg/ml of FSL-1 and 1% chitosan composite preservative (FCCP-1, FCCP-2, FCCP-3, and FCCP-4), and the FSL-1 concentrations were analyzed the effect of FSL-1 concentration on the physicochemical properties of the composite preservatives, including their film-forming ability, antioxidant capacity and ability to prevent bacterial growth, was analyzed. To further investigate the effect of the combined preservatives on fresh-cut lettuce, different FCCPs were applied to the surface was stored at 4°C for 7 days. Then the changes in weight loss, hardness, browning index, total chlorophyll content, SOD and MDA were analyzed. It was used to assess the physicochemical indicators of fresh-cut lettuce throughout storage. According to the results of Fourier transform infrared spectroscopy, FSL-1 and chitosan interacted to form hydrogen bonds, and the contact angle and viscosity of FCCP increased on both horizontal glass and polystyrene plates, indicating the good film-forming properties of the composite preservation solution. With the diameter of the antibacterial zone of *Staphylococcus aureus*, *Escherichia coli*, *Salmonella typhimurium*, and *Listeria monocytogenes* being (21.39 ± 0.22), (17.43 ± 0.24), (15.30 ± 0.12), and (14.43 ± 0.24) mm, respectively. It was proved that the antibacterial activity of FCCP became stronger with the increase of FSL-1 concentration and had the best antibacterial effect on *S. aureus*. The complex preservative showed the best scavenging effect on ferric reducing antioxidant capacity, DPPH radicals (96.64%) and 2,2’-Azinobis- (3-ethylbenzthiazoline-6-sulphonate) (ABTS) radicals (99.42%) when FSL-1 was added at 2 mg/ml. When fresh-cut lettuce was coated with FCCP for the same storage time, various indicators of lettuce such as weight loss, hardness, browning index, SOD activity and MDA content were better than the control group showing good potential in fresh-cut vegetables and fruits preservation. FCCP holds great promise for food safety quality and shelf-life extension as a new natural food preservative. The waste utilization of sea buckthorn leaves can greatly improve his utilization and economic benefits.

## 1. Introduction

A deciduous shrub or tree belonging to the genus *Hippophaerhamnoides* in the family *Elaeagnaceae* is known as *Hippophaerhamnoides Linn* (sea buckthorn; Ciesarová et al., 2020). Due to its quick growth and strong root structure, which can withstand wind and sand and lessen soil erosion, sea buckthorn offers excellent ecological advantages. The SBL’s significant therapeutic potential is due to its abundance of active components, which include proteins, polysaccharides, organic acids, alkaloids, flavonoids, amino acids, carotenoids, chlorophyll, and trace minerals (Michel et al., 2012; Li et al., 2021b). However, a lot of academics and businesspeople focus more on the study and application of its fruits and seeds while ignoring the study and advancement of sea buckthorn leaves (SBL), leading to a significant loss of sea buckthorn by-product resources. In addition, SBL has the highest concentration of flavonoids of all sea buckthorn parts (Cho et al., 2014), and flavonoids of sea buckthorn leaf (FSL) are primarily known for their pharmacological effects, which include antioxidant, antibacterial, aging-delaying, cardiovascular disease-preventing, enhancing immune function, regulating blood sugar and blood lipids, and inhibiting tumor growth. According to the research, the extract of FSL in the Gansu area mainly contains rutin, kaempferol, quercetin, isorhamnetin, and prunetin, and the contents of prunetin and isorhamnetin were significantly higher than those of seeds and fruits, and prunetin has various pharmacological activities such as anti-inflammatory and analgesic, anti-tumor, hypoglycemic and hepatoprotective, while isorhamnetin has pharmacological effects of hypoglycemic and lipid-lowering activities (Hui et al., 2017). These properties of FSL are important for enhance the antibacterial activity and antioxidant activity of active food packaging films are highly desirable, but their application in the latter has not been explored. As the safety of synthetic antioxidants has been questioned, the search for safe and efficient natural antioxidants has become an important research direction for food additives (Pundir et al., 2021).

By including antioxidants, antimicrobial agents, or other active ingredients, compound preservation agents are a novel way to preserve food (Chaudhary et al., 2020). Adding antioxidants, antimicrobial agents, or other active substances to a compound preservation agent can extend the shelf life of food. Deacetylated chitin, often known as chitosan (CS), is a naturally occurring polysaccharide that is obtained from crab and shrimp shells. It is excellent as a substance for a food preservation agent since it forms nice films, is biodegradable, antimicrobial, and safe (Talón et al., 2017). A compound preservative’s effect is preferable to a single natural preservative’s poor performance. Fresh-cut fruits and vegetables are frequently preserved with chitosan compound. Everyone enjoys *LactucasativaL* (lettuce), which is also known as green leaf lettuce and is nutritious, fresh, and crisp. Fresh-cut lettuce, however, experiences mechanical damage as well as a number of unfavorable physiological and biochemical changes, including surface browning and wilting, microbial infestation, and nutrient loss, which reduce sensory quality, commercial value, and shelf life. Studies have shown that film coating treatment can limit the loss of chlorophyll and ascorbic acid, delay the appearance of browning, and inhibit the increase in weight loss rate and decrease in hardness of fresh-cut fruit and vegetable lettuce.

In this paper, we first analyzed the optimal extraction conditions and purification method of FSL, and prepared an antibacterial and antioxidant FSL-1 and chitosan compound preservative (FCCP) combined with chitosan. The physical properties of FCCP, including infrared absorption spectra, viscosity, and contact angle, were then evaluated. The potential of FCCP for fresh-cut lettuce preservation was further evaluated, including measurement of weight loss, browning index, hardness, chlorophyll content, superoxide dismutase activity (SOD) and malondialdehyde content (MDA), to provide a research basis and direction for the development and utilization of FSL-1 in the pharmaceutical, food and nutraceutical industries, so that SBL can be turned into treasure to a greater extent and the added value of sea buckthorn resources can be improved.

## 2. Materials and methods

### 2.1. Materials

Sea buckthorn leaves (Gansu Huipeng Herb Company, China), *Staphylococcus aureus* (*S. aureus*; GDMCC1.12442), *Escherichia coli* (*E.coli*; GDMCC 1.173), *Salmonella typhimurium* (*S. typhimurium*; GDMCC 1.1442), *Listeria monocytogenes* (*L. monocytogenes*; GDMCC1.2408; Guangdong Microbial Strain Conservation Center, China); chitosan (90% deacetylation degree; Shanghai Yuanye Biotechnology Co., Ltd., China); lettuce (Chengdu Emerald Mountain Treasure Agriculture Co., Ltd., China); SOD kit, MDA kit and protein kit (BCA method; Nanjing Jiancheng Institute of Biological Engineering Co., Ltd., China); Vitamin C (V_C_; Shanghai Yuanye Biotechnology Co., Ltd., China); LB broth agar medium (Shanghai Yuanye Biotechnology Co., Ltd., China).

Sea buckthorn leaves was authenticated by Zhang Runrong, senior engineer of traditional Chinese medicine, College of Pharmacy and Food Science, Zhuhai College of Science and Technology, Zhuhai, Guangdong Province, China.

### 2.2. Extraction and purification of FSL-1

The optimal extraction conditions of sea buckthorn leaf flavonoids were obtained by single-factor and response surface experiments. The appropriate amount of defatted SBL powder was weighed and sieved to 0.18 mm, and FSL was extracted by Ultrasonic Extractor (XH-300A, XiangHu Technologies, China) with 47% ethanol in the ratio of 1:19. The extraction time was set to 34 min, and the extraction temperature was 65°C. After freeze-drying at −40°C using vacuum freeze–dryer (FD-250101, FTFDS, China), store it in −20°C refrigerator, the FSL was centrifuged at room temperature for 10 min (4,000 rpm/min), transferred the supernatant, concentrated and lyophilized to obtain FSL crude extracts (Culina et al., 2021).

**Figure 1:**
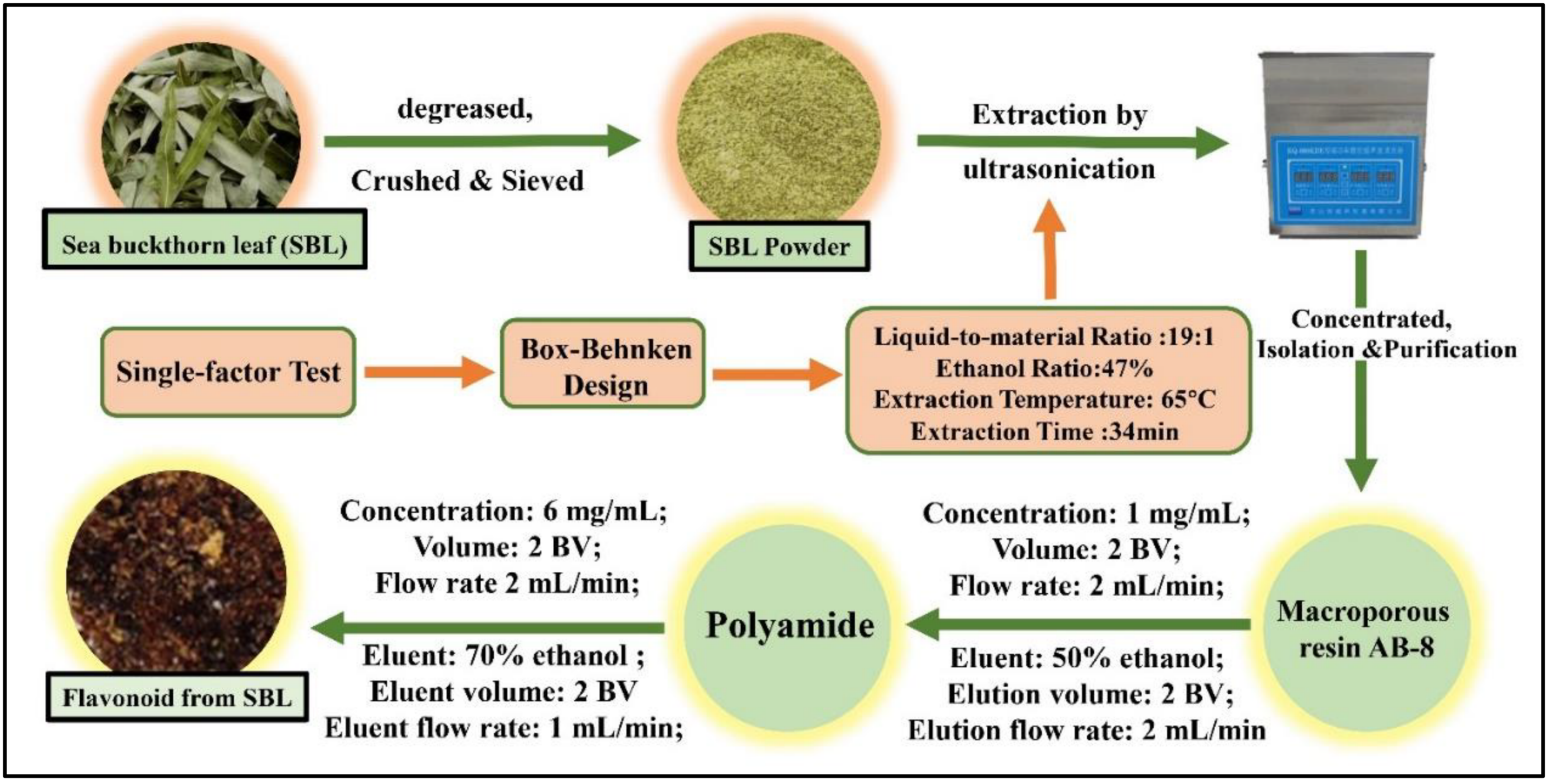
The extraction and purification procedure of FSL-1.

In the purification, the crude FSL were separated and purified by using macroporous resin AB-8, and then the FSL was purified for the second time by using polyamide, and the eluate was lyophilized to obtain the purified flavonoids from SBL (FSL-1), which was stored in the refrigerator at −20°C (Wan et al., 2014). The extraction, isolation, and purification procedure of FSL-1 is shown in Figure 1.

### 2.3. Content identification of FSL

The standard control was selected as rutin, and the sample solution was prepared into concentration gradients of 0.02, 0.04, 0.06, 0.08, 0.10, 0.12 mg/ml, and the absorbance values were determined according to the Al(NO3)_3_–NaNO_2_ colorimetric method (Yuan-yuan et al., 2011), which was selected at 500 nm. The extracted sample solution was diluted into each suitable concentration gradient, and the determination was carried out with reference to the method of standard curve, and then the content of flavonoids (mg/mL) in SBL flavonoids extract was determined quickly according to the conversion of standard curve. The content of total flavonoids in SBL was calculated according to the following formula:


$$\text{FSL}\;\text{content}\left(\%\right)=\frac{\text{cvd}}{m}\times 100.$$

C: flavonoid concentration of SBL (mg/mL); V: sample volume; D: dilution multiple; m: mass of SBL (g).

### 2.4. Preparation of FCCP

Different concentrations of FSL-1 were dissolved in 1.0% chitosan solution and agitated with constant magnetic force (1800 r/min) for 1 h at room temperature 25°C with ultrasonic shaking for 15 min to obtain flavonoid of sea buckthorn leaf and chitosan complex preservative (FCCP) and set aside. The FCCP was divided into five groups for experiments include 1.0% chitosan preservative (CP), 0.5 mg/mLFSL-1 + 1.0% chitosan preservative (FCCP-1), 1.0 mg/mLFSL-1 + 1.0% chitosan preservative (FCCP-2), 1.5 mg/mLFSL-1 + 1.0% chitosan preservative (FCCP-3), and 2.0 mg/mLFSL-1 + 1.0% chitosan preservative (FCCP-4) was mixed well in proportion to obtain FCCP.

### 2.5. Determination of Fourier transform infrared spectroscopy

The fully dried KBr and flavonoid samples FSL-1 and FCCP were ground in a dry environment at a mass ratio of 100:1 ~ 200:1, mixed thoroughly with an onyx mortar, pressed and then scanned with a FTIR spectrometer (Spectrum 3, Perkin Elmer, United States) in the range of 400 cm^−1^ ~ 4,000 cm^−1^ (Hu et al., 2016).

### 2.6. Determination of coating properties of FCCP solution

Chitosan film solution (40 μl) was dropped onto a clean and flat glass plate and a polystyrene plastic plate (2.6 × 7.6 cm^2^), and the contact angle size of the film solution on the surface of two different contact substrates at room temperature 25°C was measured by Contact angle measuring instrument (SL200L2, Shanghai Soren Information Technology Co., Ltd., China).

Chitosan film solution (10 ml) was added into the Digital Viscometer (SNB-2, Shanghai Jingtian Electronic Instruments Co., Ltd., China) and the viscosity of FCCP was measured at room temperature of 25°C.

### 2.7. Determination of antibacterial performance test

The diameter of the antibacterial zone using the punching method was used to assess the antibacterial activity of FCCP against *S. aureus*, *E. coli*, *S. typhimurium*, and *L. monocytogenes*, four food-borne pathogens. The bacterial strains were first incubated in nutrient broth at 37°C for 24 h. Then, 0.2 ml of 10^6^ CFU/ml bacterial culture was added and dispersed uniformly on LB agar plates. A circular membrane (1 cm in diameter) was placed on the plate and the bacteria were incubated at 37°C for 12 h. The diameter of the antibacterial zone was measured by vernier calipers.

### 2.8. Determination of DPPH free radical scavenging activity

The DPPH free radical scavenging activities of the samples were tested according to a reported (Genskowsky et al., 2015) procedure with some modifications. Briefly, the DPPH test solution was first diluted with methanol to 0.6 mM to obtain an absorbance of 0.7–0.9 at 517 nm. The stock solutions of FSL-1 (100 μl) and FCCP (100 μl) were continuously diluted in half with distilled water on a 96-well plate separately, and reacted with 100 μl of DPPH radical solution for 30 min in dark. The absorbance of the reaction solution and the initial DPPH radical solution diluted with distilled water were measured with a UV–vis reader at 517 nm. V_C_ was used as the positive control. When FCCP mass concentration is 0, it is chitosan alone. The antioxidant capacity of the FSL-1 and the FCCP coating solutions was calculated by the following equation:


$$\text{scavenging rate}\;\left(\%\right)=\frac{A_{0}-A_{1}}{A_{0}}\times 100\%.$$

where *A*_0_ is the absorbance of the DPPH radical solution (100 μl) at 517 nm, *A*_1_ is the absorbance of the reaction solution at 517 nm.

### 2.9. Determination of ABTS free radical scavenging activity

The ABTS scavenging activities of the samples were tested according to a reported procedure with some modifications (Re et al., 1999). The ABTS radical cation was generated by reacting 10 ml of 7 mM ABTS stock solution with 10 ml of 2.45 mM potassium persulfate solution in the dark at room temperature overnight. The resulting mixture was diluted to obtain a stock solution with an absorbance of 0.7 ± 0.005 units at 734 nm. V_C_ was used as the positive control. The same procedure for the ABTS was used to determine the antioxidant performance of FSL-1 and FCCP to ABTS free radicals using the same as the formula in 2.8.

### 2.10. Determination of ferric-reducing antioxidant power

The method of ferric-reducing antioxidant power was determined by referring to the method with some modifications (Tang et al., 2014). 1 ml of flavonoids samples were added into 2.5 ml PBS (pH = 6.6) and 2.5 ml 1% potassium ferricyanide solution. Put it in a water bath at 50°C for 20 min, add 2.5 ml of 10% trichloroacetic acid and shake well. Centrifugation at 3,000 r/min for 10 min. Take 2.5 ml supernatant, add 0.5 ml 0.1% ferric chloride solution and 2.5 ml distilled water, and shake well. The absorbance of the reaction solution measured at 700 nm with Microplate Reader (Epoch, Bio Tek Instruments Inc, United States) was denoted as *A*_i_ and that of the control group was denoted as *A*_0_ with distilled water instead of the sample. V_C_ was used as the positive control. The higher the absorbance value indicates the stronger the total reducing ability.

### 2.11. Application for storing fresh-cut lettuce

Lettuce was washed with 100 mg/l sodium hypochlorite solution, dried in a sterile operation table at room temperature of 25°C, then cut into 1 cm^3^ and immersed in different preservation solutions (CP, FCCP-1, FCCP-2, FCCP-3, and FCCP-4) for 10 min, and stored in a 4°C refrigerator for 7 days after film formation at natural temperature, and data such as weight and BI were measured every 24 h. Distilled water was used instead of the preservation solution as a control.

### 2.12. Determination of weight loss rate

The following formula was used to calculate the weight loss of fresh-cut lettuce:


$$\text{Weight loss rate}\;\left(\%\right)=\frac{W_{1}-W_{i}}{W_{1}}\times 100\%,$$

where *W*_1_ and *W*_i_ are the weight of fresh-cut lettuce at 0 day and *n*th days, respectively.

### 2.13. Determination of hardness

The hardness of fresh-cut lettuce was measured using a mass spectrometer (TA.TOUCH, Shanghai Baosheng Company, China) with a probe diameter of 5 mm and the following parameters: trigger force 0.4 N; detection rate 1.5 mm/s; post-measurement rate 3 mm/s; compression distance 2.5 mm (Rinaldi et al., 2019).

### 2.14. Determination of browning index

BI was used to quantify the browning degree of fresh-cut lettuce during storage. The color parameters (L*, a*, and b*) of the samples were measured using a colorimeter (CR-10 PLUS, Konica Minolta, Tokyo, Japan). The BI of fresh-cut lettuce was calculated as follows:


$$\text{BI}=\sqrt{{\left(\Delta L^{*}\right)}^{2}+{\left(\Delta a^{*}\right)}^{2}+{\left(\Delta b^{*}\right)}^{2}}$$

Where $\Delta L^{*}$, $\Delta a^{*},$ and $\Delta b^{*}$are the change of fresh-cut lettuce at 0 day to 7 days. $L^{*}$ represents the black and white value, $a^{*}$ represents the red and green value, $b^{*}$ represents the yellow and blue value.

### 2.15. Determination of total chlorophyll content

After 7 days of storage at 4°C, 1 g of each lettuce was weighed and placed in a pre-cooled mortar using 80% acetone grinding method, 5 ml of extract (80% acetone) and a small amount of quartz sand were added and ground to a homogeneous form, filtered through 7 cm filter paper, washed with extract and fixed to 25 ml, and the absorbance values were measured immediately at 645 and 663 nm (Costache et al., 2012). The chlorophyll content of lettuce was calculated as shown below:


$$\begin{array}{l} \text{Total chlorophyll content}(\text{mg}\cdot g^{-1})\\ =\left[(8.04\times A_{663}+20.29\times A_{645})\times V\right]/m. \end{array}$$

*A*_663_, absorbance at 663 nm; *A*_645_, absorbance at 645 nm; *V*, total volume of chlorophyll extracts (mL); *m*, weight of the sample (g).

### 2.16. Determination of SOD activity and MDA concentration

First-day fresh-cut lettuce as a control and fresh-cut lettuce stored at 4°C for 7 days were made into homogenates and measured and calculated according to the appropriate kit instructions (Li et al., 2021a).

### 2.17. Statistical analysis

Data are shown as mean ± SEM, and statistical analysis was performed using Origin 2021, GraphPad Prism 8, and EXCEL 2019. To compare the differences between multiple groups, a one-way analysis of variance (ANOVA) was performed in this software. *p < 0.05* indicates statistical significance.

## 3. Results

### 3.1. Analysis of extraction and purification contents

The FSL content was 66.89 mg/ml by ultrasonic extraction, and then the FSL purity was increased from 43.00 to 62.25% by the separation and purification of FSL with the choice of macroporous resin AB-8, and then the second purification of FSL with the choice of polyamide resin, under which the sample was a purple-black powder and the purity of FSL was increased to 80.75%. After the above steps, FSL-1 was eventually available.

### 3.2. Analysis of FT-IR spectroscopy

**Figure 2:**
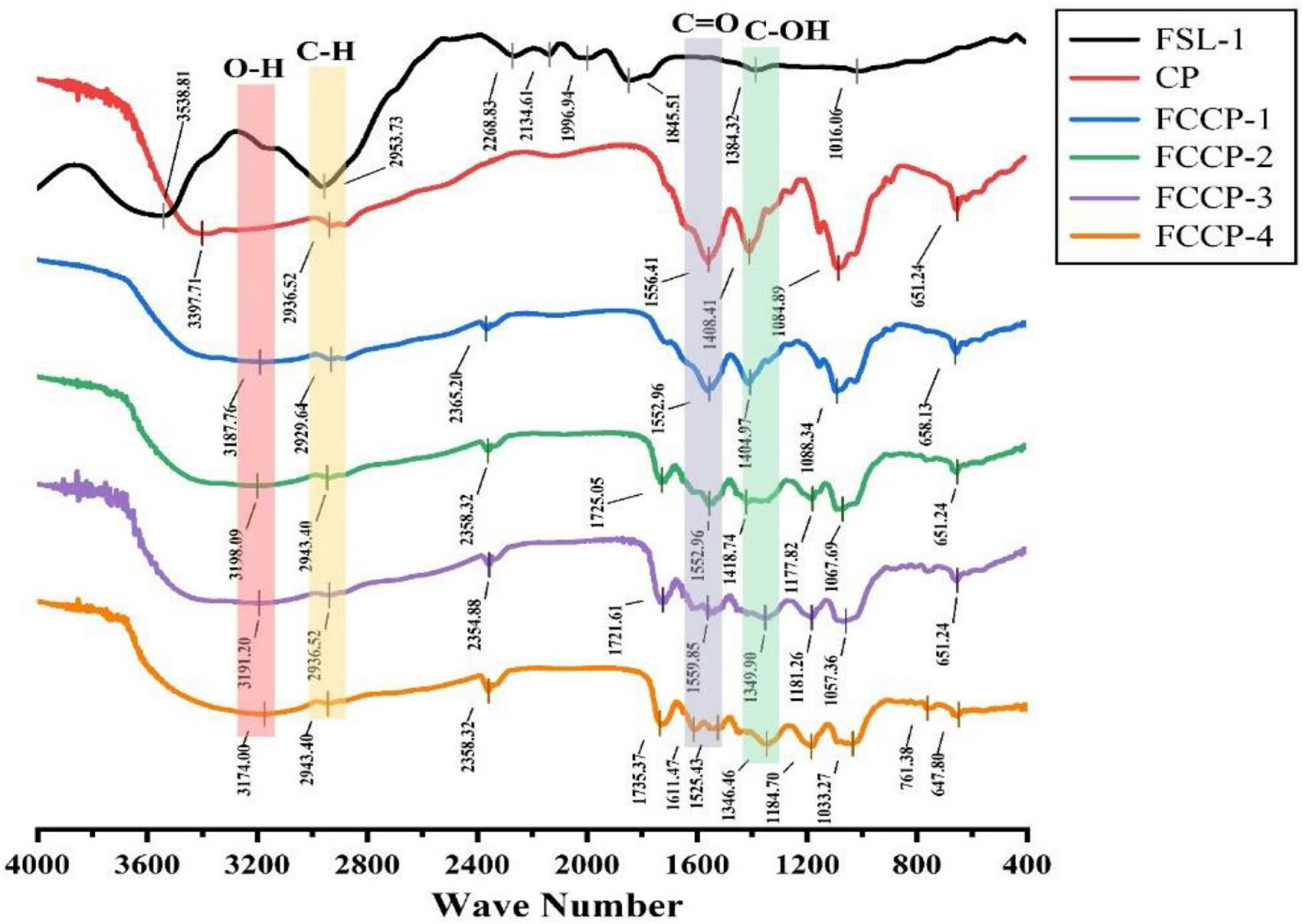
The results of Fourier transform infrared (FT-IR) spectroscopy of FSL-1, CP, FCCP-1, FCCP-2, FCCP-3, and FCCP-4.

The parent nuclei of flavonoids contain hydroxyl, phenolic hydroxyl, methoxy, alkoxy, benzene and other groups with infrared characteristic absorption peaks at 3,100–3,460 cm^−1^, 1,600–1,640 cm^−1^, and 1,380 cm^−1^, 1,260 cm^−1^, 1,090 cm^−1^. As can be seen from Figure 2 (FSL-1), the broad and strong absorption peaks at 3,385 cm^−1^ wave number indicate the presence of a large number of phenolic hydroxyl groups; the characteristic absorption peaks at 2,917 cm^−1^, 2,853 cm^−1^, 1,380 cm^−1^ are –CH3, –CH2, indicating the presence of hydrogen on saturated carbon; the characteristic peaks at 1,630, 1,610, 1,540, and 1,450 cm^−1^ are benzene ring peaks. The absorption peaks at 1670 cm^−1^ are C = 0 stretching vibration peaks, in which the group may be conjugated with the hydroxyl group causing its absorption peak to move to lower wave number, and at 1,260 and 1,090 cm^−1^ can be attributed to the antisymmetric and symmetric stretching vibration peaks of the ether bond, Analysis results indicate that the extract is a flavonoid (Agatonovic-Kustrin et al., 2021).

The IR spectrograms of different membranes were measured to confirm the intermolecular interactions between membrane components. As shown in Figure 2, the peak of CP at 3,254 cm^−1^ corresponds to the O–H and N–H stretching vibrations of CS and the O–H stretching vibration of the FSL-1 fraction in FCCP, the peak at 2,869 cm^−1^ corresponds to the C–H stretching vibration of CS and FSL-1, the peak at 1,630 cm^−1^ corresponds to the C = 0 stretching vibration of amide I in CS The peak at 1,549 cm^−1^ corresponds to the N–H stretching vibration of CS, the peak at 1,406 cm^−1^ corresponds to the C–H stretching vibration of CS and FSL-1, and the peak at 1,024–1,150 cm^−1^ corresponds to the pyranose ring stretching vibration of CS (Bi et al., 2020). The IR spectrograms of CP, FCCP-1, FCCP-2, FCCP-3, and FCCP-4 changed slightly after the addition of flavonoids. FCCP-1, FCCP-2, FCCP-3, and FCCP-4 showed wider and stronger O–H and N–H stretching vibrational bands at 3,223–3,270 cm^−1^ than CP, which was attributed to the large amount of hydroxyl groups in flavonoids. Compared with CP, the films containing flavonoids were red-shifted or blue-shifted at 3,223–3,270 cm^−1^ (O–H and N–H stretching bands) and 1,535–1,549 cm^−1^ (N–H stretching bands; Kaya et al., 2018). The band shifts were mainly caused by intermolecular hydrogen bonds formed between the hydroxyl groups of flavonoids and other membrane components (CS). Similar band shifts were also observed in other flavonoid-rich membranes due to the formation of intermolecular interactions. However, in the inclusion compound, the characteristic peaks with wave numbers of 1692.28 cm^−1^ (C=C stretching vibration) and 1509.23 cm^−1^ (C=O stretching vibration) disappeared, which led to the speculation that the C ring of sea buckthorn flavonoids might be encapsulated within the CS (Li et al., 2016; Artiga-Artigas et al., 2018).

### 3.3. Analysis of coating properties of FCCP solution

**Figure 3:**
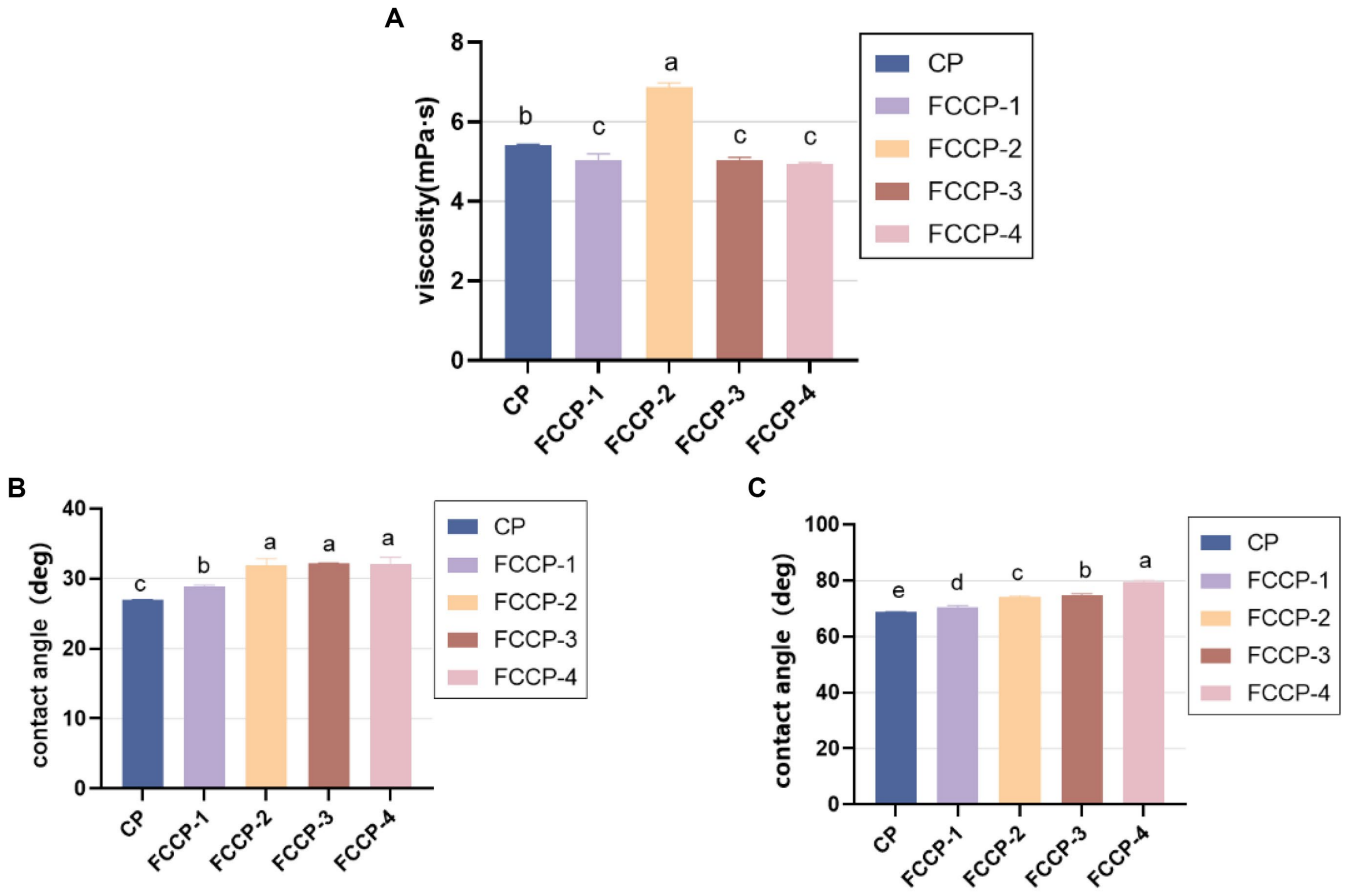
**(A)** Viscosity of FSL-1, CP,FCCP-1,FCCP-2,FCCP-3 and FCCP-4. **(B)** Contact angles of FSL-1,CP,FCCP-1,FCCP-2,FCCP-3 and FCCP-4 in glass plates. **(C)** Contact angles of FSL-1, CP, FCCP-1,FCCP-2,FCCP-3 and FCCP-4 on polystyrene plates.

The film-forming effect of chitosan is closely related to the flow and deformation of the film solution, so it is important to study the rheological behavior of the film solution for the quality control of the compound preservative. The viscosities of different FCCP as shown in Figure 3A, as the concentration of FSL-1 increases, the viscosity of FCCP decreases, then increases and then decreases, and the viscosity of FCCP-2 reaches the maximum and FCCP-4 reaches the minimum, with a difference of 28%.

Fruit preservatives should have a certain water retention capacity. This is because fruits will lose water and shrink during storage. Therefore, we measure the hydrophilicity of the coating by contact angle. For more effective preservative purposes, the chitosan solution should have excellent wettability on both hydrophilic and hydrophobic surfaces. As a basic indicator of wettability, the contact angles of FCCP on horizontal glass and polystyrene plates are shown in Figures 3B,C, respectively. The contact angles on both glass plates are less than 40°, indicating that FCCP easily diffuses on hydrophilic surfaces, while the contact angles on polystyrene substrates range from 70° to 80°. The contact angles on both substrates increase with increasing FSL-1, which means that FCCP wets worse on the glass plate versus the polystyrene plate. The contact angle reflects the water resistance of the film surface, the larger the contact angle, the stronger the hydrophobicity, when the contact angle > 65°, it means that the film has a hydrophilic surface with certain water resistance, on the contrary, it means that the film surface is extremely hydrophilic. The contact angle of CP was 68.4°, but after adding FSL-1, the contact angle increased up to 11°, probably due to the interaction between FSL-1 and CS, which made the hydrophobicity stronger. As the concentration of FSL-1 increased, the surface roughness of the composite membrane increased due to the increase of surface-active ingredients, which caused the contact angle of the composite membrane to increase gradually and the hydrophilicity to decrease gradually. Overall, the contact angle of all groups of films showed hydrophilic (*θ* < 90°), which is suitable for low moisture food packaging (Figure 3).

### 3.4. Analysis of antibacterial zone

According to Table 1, the size of the diameter of the antibacterial zone against foodborne pathogenic bacteria. It can be seen that FCCP has inhibitory effect on all four foodborne pathogenic bacteria, but there are obvious individual differences in the tolerance of the four pathogenic bacteria to FCCP. Under the same conditions, CP also had a certain antibacterial effect, but the antibacterial zone boundary was not obvious enough compared with other FCCP added with different concentrations of FSL-1. The antibacterial effect of FCCP on the four bacteria was better than that of CP, and the antibacterial effect of FCCP-2 was stronger as the concentration of FSL-1 increased. A strong antibacterial effect was obtained when the antibacterial cycle diameter was 21.39 ± 0.22 mm for *S. aureus*, 17.43 ± 0.24 mm for *E. coli*, 15.30 ± 0.12 mm for *S. typhimurium* and 14.43 ± 0.24 mm for the *L. monocytogenes*. While the strongest FCCP-2, which showed the strongest activity for *Sauers* and showed the weakest activity for *Limnocyonines*. It demonstrates that the compound’s antibacterial properties are optimal and that it can better preserve freshly cut fruits and vegetables.

**Table 1:** Antibacterial zone of chitosan preservative (CP), FCCP-1, FCCP-2, FCCP-3 and FCCP-4.

Grouping	Antibacterial zone (mm)			
	*Staphylococcus aureus*	*Escherichia coli*	*Salmonella typhimurium*	*Listeria monocytogenes*
Control	NE	NE	NE	NE
CP	20.26 ± 0.23^c^	15.25 ± 0.45^c^	13.83 ± 0.21^c^	12.04 ± 0.50^b^
FCCP-1	20.53 ± 0.21^bc^	16.38 ± 0.31^b^	14.18 ± 0.26^b^	14.09 ± 0.16^a^
FCCP-2	20.74 ± 0.11^bc^	16.47 ± 0.32^b^	14.30 ± 0.28^b^	14.18 ± 0.27^a^
FCCP-3	20.78 ± 0.45^b^	16.62 ± 0.38^ab^	14.30 ± 0.44^b^	14.30 ± 0.28^a^
FCCP-4	21.39 ± 0.22^a^	17.43 ± 0.24^a^	15.30 ± 0.12^a^	14.43 ± 0.24^a^

“NE” It means no bacteriostatic effect. Each value represents mean ± SD of triplicates. Different lower-case letters in the same bacterial strain indicate significantly different (*p* < 0.05).

### 3.5. Analysis of DPPH free radical scavenging activity

**Figure 4:**
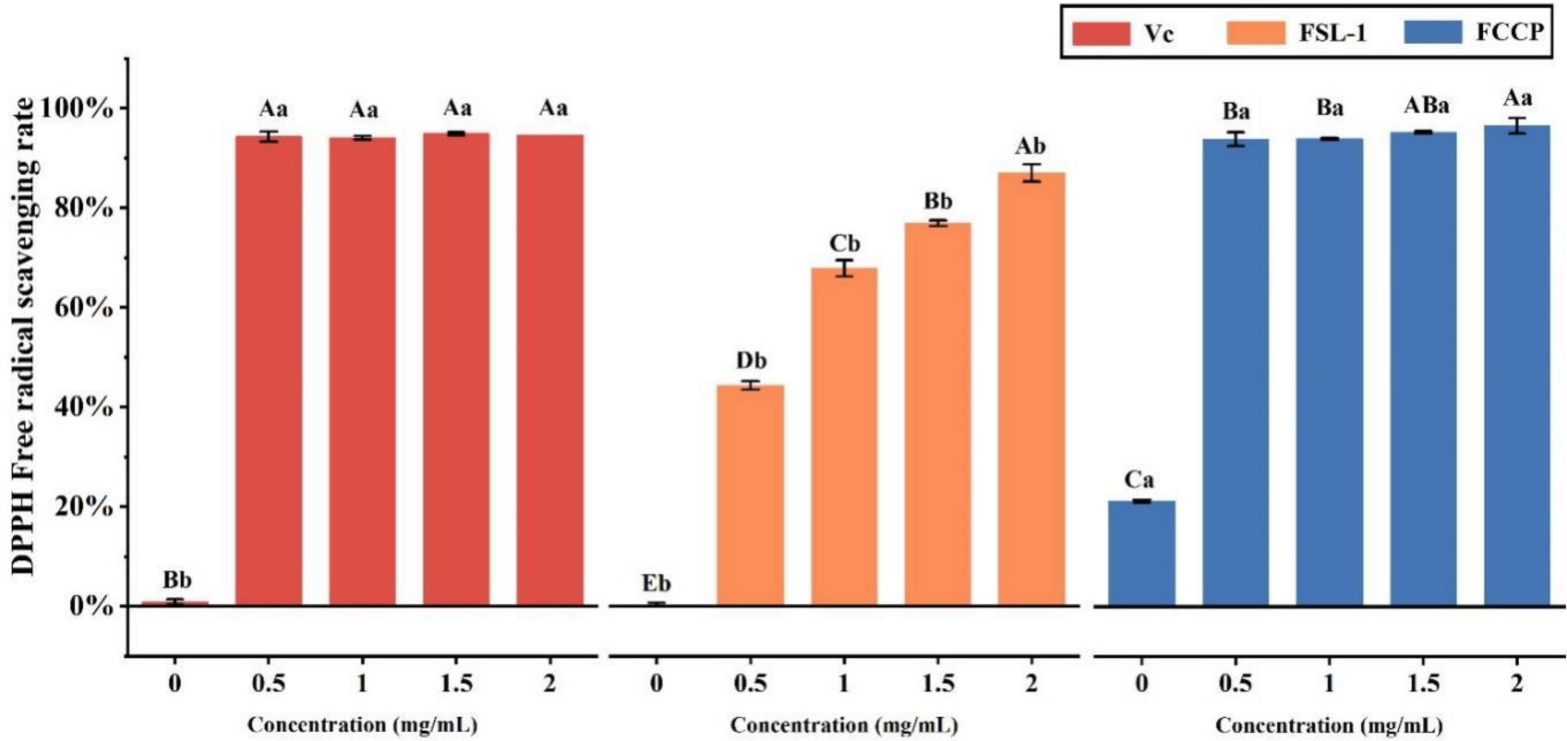
DPPH free radical scavenging activity of V_C_, FSL-1 and FCCP. If containing an identical lowercase (uppercase) letter is not a significant difference, where no identical lowercase (uppercase) letter is a significant difference (*p* < 0.05).

Domestic and international studies have shown that FSL-1 has optimum antioxidant effects. As demonstrated in Table 2 and Figure 4. 0.5 mg/ml scavenging rate tended to be stable; while FSL-1 scavenging rate was stable at about 87% at mass concentrations greater than the DPPH radical scavenging ability of FCCP at a concentration of 2.0 mg/ml was higher than that of the positive control V_C_ at the same concentration, with a maximum scavenging rate of about 97%. The scavenging rate of chitosan preservative alone was only 18.74%, indicating that FSL-1 can greatly improve the antioxidant performance of the composite preservative with the increase of content.

**Table 2:** DPPH (1, 1-Diphenyl-2-picrylhydrazyl radical; 2,2-Diphenyl-1-(2,4,6-trinitrophenyl)hydrazyl; α,α-Diphenyl-β-picrylhydrazyl radical. free radical scavenging activity of V_C_, FSL-1, and FCCP.

Mass concentration (mg/mL)	DPPH radical scavenging activity (%)		
	V_C_	FSL-1	FCCP
0	1.97 ± 1.61^Bb^	0.67 ± 1.02^Eb^	18.74 ± 5.22^Ca^
0.5	94.13 ± 0.36^Aa^	45.78 ± 2.22^Db^	93.92 ± 1.43^Ba^
1	94.35 ± 1^Aa^	67.95 ± 1.65^Cb^	93.99 ± 0.21^Ba^
1.5	94.92 ± 0.29^Aa^	76.97 ± 0.57^Ab^	95.3 ± 0.24A^Ba^
2	94.64^Aa^	87.12 ± 1.72^Ca^	96.64 ± 1.57^Aa^

The upper and lower case letters indicate the results of the different group comparisons.

### 3.6. Analysis of ABTS free radical scavenging activity

**Figure 5:**
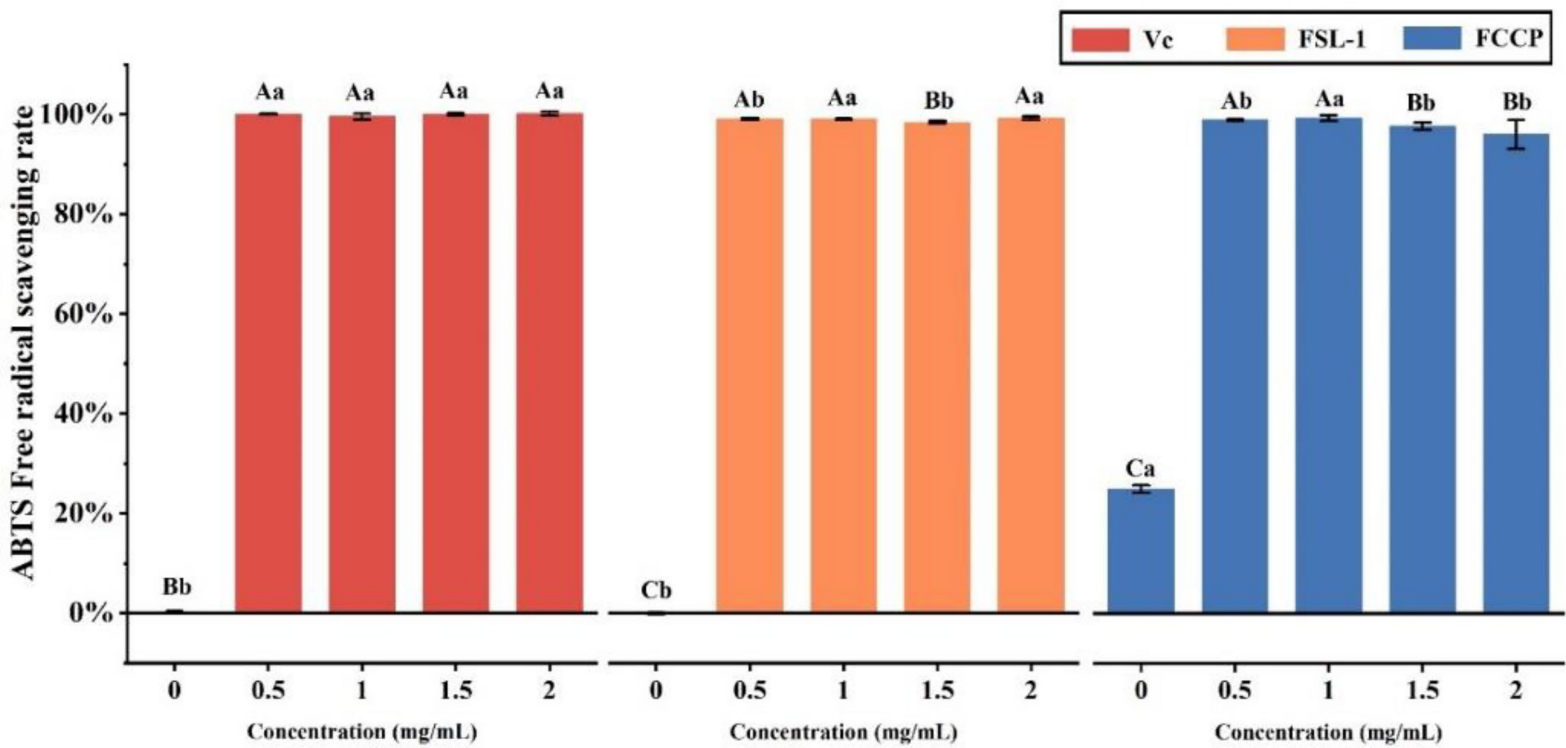
ABTS free radical scavenging activity of V_C_, FSL-1 and FCCP. If containing an identical lowercase (uppercase) letter is not a significant difference, where no identical lowercase(uppercase) letter is a significant difference (*p* < 0.05).

The total antioxidant capacity was determined by the ABTS method, Table 3 and Figure 5 shows that the mass concentrations of FSL-1, FCCP and positive control V_C_ were positively correlated with the total antioxidant capacity at mass concentrations of 0–2.0 mg/ml, and the higher the mass concentration, the stronger the total antioxidant capacity. The ABTS radical scavenging rate of chitosan preservative alone was only 25%, though the total antioxidant capacity of both FSL-1 and FCCP was lower than that of the positive control V_C_ under the same mass concentration conditions. The scavenging rate of ABTS radicals was stabilized at 99.42% after FCCP reached a mass concentration of 2.0 mg/ml. The decrease in the total antioxidant capacity of FCCP might be due to the high concentration of FSL-1, which led to a decrease in the total antioxidant capacity of the compound film solution due to the longer resting time during the process of flow forming. Longer, part of FSL-1 precipitated out in the form of flocculent, and the content of TP active substances in the compound film decreased, thus affecting the capturing ability of the polyphenolic compounds in the compound film for DPPH radicals.

**Table 3:** ABTS free radical scavenging activity of V_C_, FSL-1, and FCCP.

Mass concentration (mg/mL)	ABTS radical scavenging activity (%)		
	V_C_	FSL-1	FCCP
0	0.16 ± 0.26^Bb^	0.78 ± 0.71^Cb^	25 ± 0.78^Ca^
0.5	99.61 ± 0.58^Aa^	99.22 ± 0.19^Aa^	97.8 ± 0.71^Ab^
1	100 ± 0.13^Aa^	99.16 ± 0.13^Aa^	96.21 ± 2.91^Aa^
1.5	100 ± 0.26^Aa^	98.51 ± 0.26^Bb^	99.03 ± 0.19^Bb^
2	100 ± 0.39^Aa^	99.35 ± 0.32^Aa^	99.42 ± 0.58^Bb^

The upper and lower case letters indicate the results of the different group comparisons.

### 3.7. Analysis of ferric-reducing antioxidant power

**Figure 6:**
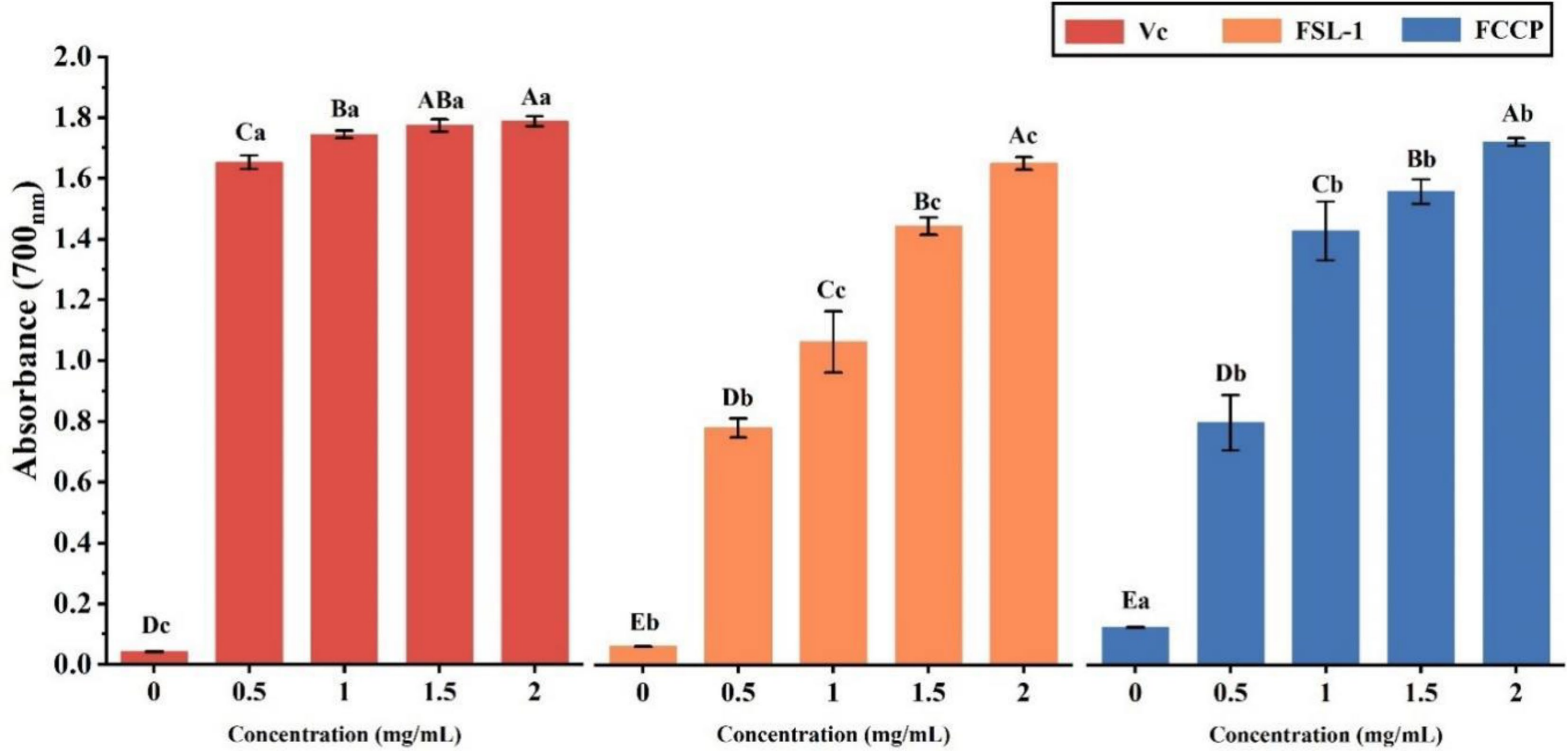
Total reducing ability of V_C_, FSL-1 and FCCP. If containing an identical lowercase (uppercase) letter is not a significant difference, where no identical lowercase (uppercase) letter is a significant difference (*p* < 0.05).

The results are shown in Table 4 and Figure 6, the ferric-reducing antioxidant power showed a positive correlation with the content of FSL-1, the higher the content of FSL-1, the higher the total reducing capacity and the difference was significant (*p* < 0.05). The total reducing capacity of FCCP was basically equal to that of the positive control V_C_, and the total reducing capacity of FSL-1 was lower than that of FCCP when and only when the mass concentration was 2.0 mg/ml, furthermore the absorbance of chitosan preservative alone was only 0.124, indicating its poor total reducing capacity, and the addition of FSL-1 improved the total antioxidant capacity of the composite preservative. And the enhancement of the total reducing capacity slowed down with the increase of mass concentration. In summary, FCCP has optimum antioxidant capacity and can be used as a natural food preservative.

**Table 4:** Total reducing ability of V_C_, FSL-1, and FCCP.

Mass concentration(mg/mL)	Total antioxidant capacity(abs)		
	V_C_	FSL-1	FCCP
0	0.043 ± 0.003^Dc^	0.06 ± 0.001^Eb^	0.124 ± 0.002^Ea^
0.5	1.654 ± 0.023^Ca^	0.779 ± 0.032^Db^	0.798 ± 0.092^Db^
1	1.745 ± 0.013^Ba^	1.063 ± 0.101^Cc^	1.429 ± 0.097^Cb^
1.5	1.774 ± 0.020A^Ba^	1.443 ± 0.028^Bc^	1.558 ± 0.040^Bb^
2	1.788 ± 0.017^Aa^	1.650 ± 0.020^Ac^	1.7215 ± 0.013^Ab^

The upper and lower case letters indicate the results of the different group comparisons.

### 3.8. Analysis of weight loss rate

**Figure 7:**
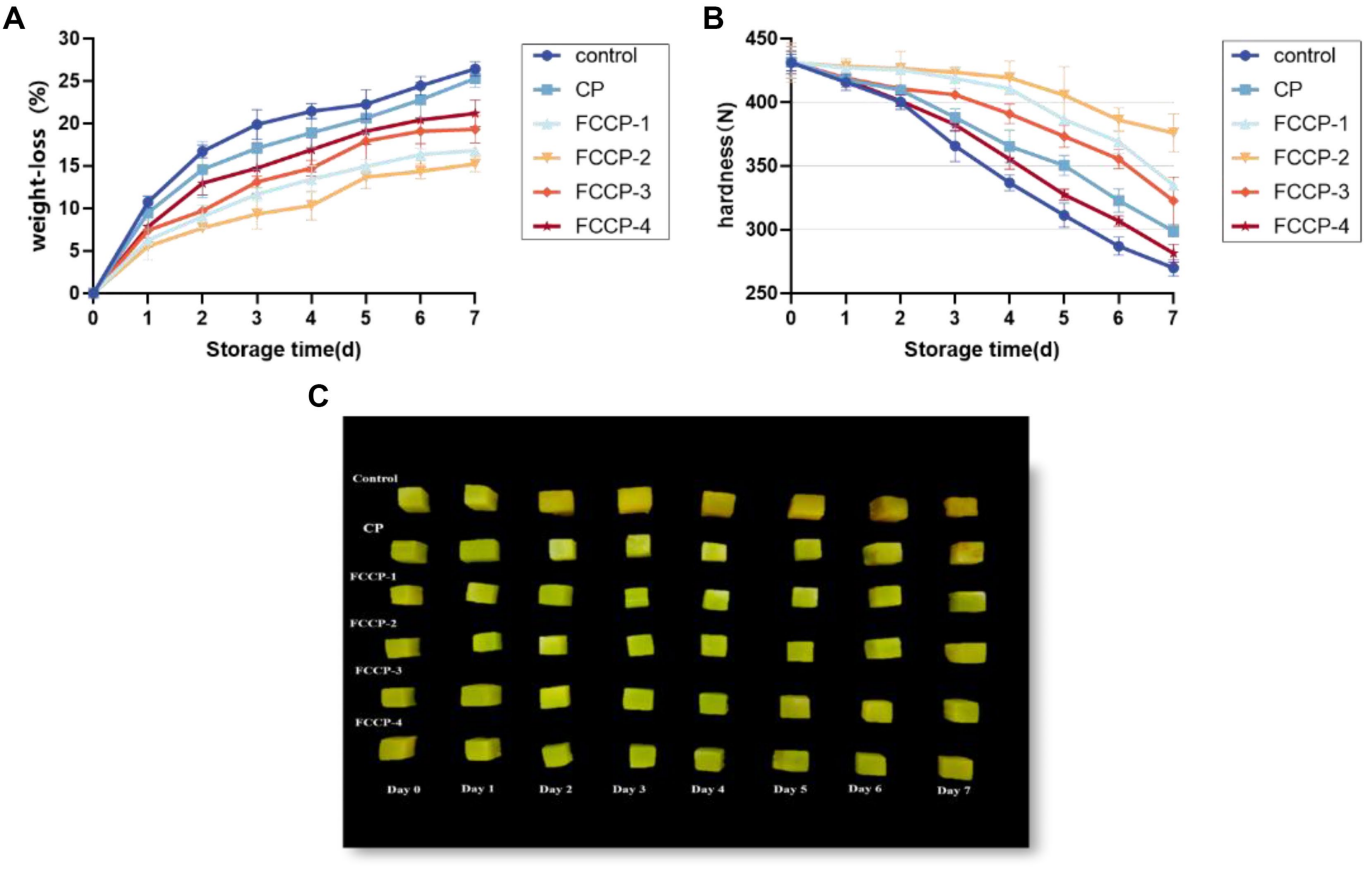
The effect of FCCP on weight loss **(A)**, hardness **(B)** and changes in the appearance **(C)** of fresh-cut lettuce stored for 7 days.

Moisture loss and nutrient depletion can lead to weight loss of fresh-cut lettuce, resulting in weight loss and loss of freshness. As shown in Figure 7A, the weight loss rate of fresh-cut lettuce during storage was on the rise, rising rapidly in the first 2 days, and the weight loss rate of the control group was nearly 25% after 7 days of storage. The weight loss rate rose slowly after FCCP treatment, which was significantly lower than that of the control group, indicating that FCCP coating treatment could effectively inhibit the weight loss of fresh-cut lettuce, of which the weight loss rate was the lowest after FCCP-2 treatment, which was 42.35% less than that of the control group.

### 3.9. Analysis of hardness

Moisture loss and destruction of cell structure can lead to a decrease in hardness and cause soft tissue distraction, so hardness is an important indicator of the sensory quality of fresh-cut lettuce. The effect of FCCP on hardness is shown in Figure 7B, the hardness of fresh-cut lettuce showed a decreasing trend during storage, and compared with the initial value, the hardness of the control group decreased by 37.38% after 7 days of storage. The hardness of the FCCP coated group was always higher than that of the control group, which indicated that FCCP could delay the decrease of the hardness of fresh-cut lettuce, in which the hardness of the FCCP-2 treated group was higher than that of the other treated groups, and the hardness changed the least before and after storage, which had the optimum effect on the maintenance of the hardness of fresh-cut lettuce. The hardness of fresh-cut lettuce was maintained optimum.

Figure 7C shows the effect of different FCCP on the quality of appearance of fresh-cut lettuce during storage. Before storage, all fresh-cut lettuce was green, and after coating, fresh-cut lettuce was yellowish in color, but all were rich in water and full of flesh. Fresh-cut lettuce without film became yellow and wrinkled after 7 days of storage, and shrunk severely due to water loss. It is worth noting that the color of fresh-cut romaine lettuce packaged with FCCP coating was only slightly darkened and there was almost no noticeable change in shape. The color change of fresh-cut lettuce during storage is consistent with the results of BI below. Compared with other FCCPs, FCCP-2 has a moderate contact angle and viscosity, so it is less hydrophilic and has better film-forming properties, which can reduce respiration and metabolism, thus reducing shrinkage and browning caused by rapid cell dehydration and cell wall hydrolysis in appearance caused by respiration and metabolism, which is beneficial for preserving cut vegetables and fruits. In addition, CG-WE films have strong antioxidant activity, which is also an important reason for inhibiting browning of fresh-cut lettuce. Thus, the above results suggest that FCCP can improve the storage quality of fresh-cut apples, while FCCP-2 showed the best performance.

### 3.10. Analysis of BI

**Figure 8:**
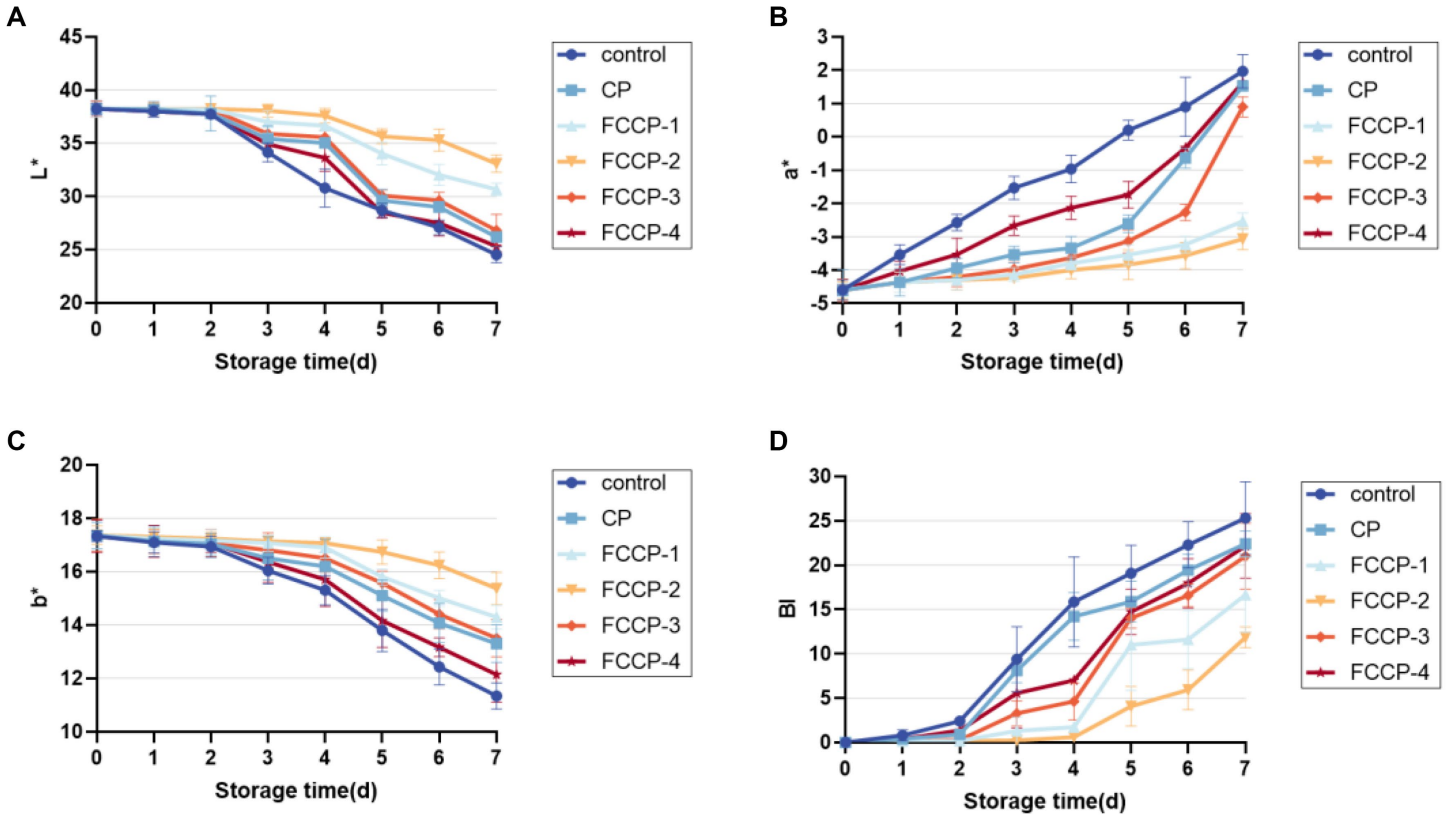
The effect of FCCP on browning index. **(A)** represents the black and white value, **(B)** represents the red and green value, **(C)** represents the yellow and blue value, **(D)** The effect of FCCP on browning index.

Lettuce is subject to tissue browning during fresh-cut processing and storage, which reduces its commercial value (Kim et al., 2014). The lettuce was green after the cutting treatment, but as the storage period was extended, the surface browned, the color darkened and the sensory quality decreased. As shown in Figure 8, the L* and b* values after FCCP treatment were higher than the control group, and the a* value and browning index were lower than the control group, indicating that FCCP treatment could inhibit the decrease of L* and b* values and the increase of a* value and browning index. FCCP helped maintain the appearance and color of fresh-cut lettuce and inhibit the aggravation of browning, among which, the browning index of FCCP-2 treatment group remained low throughout the storage period, and the browning after 7 days. The browning index of the FCCP-2 treatment group remained low throughout the storage period, and the browning index was 53.15% lower than that of the control group after 7 days, which was better than the other groups in inhibiting browning of fresh-cut lettuce.

### 3.11. Analysis of total chlorophyll content

**Figure 9:**
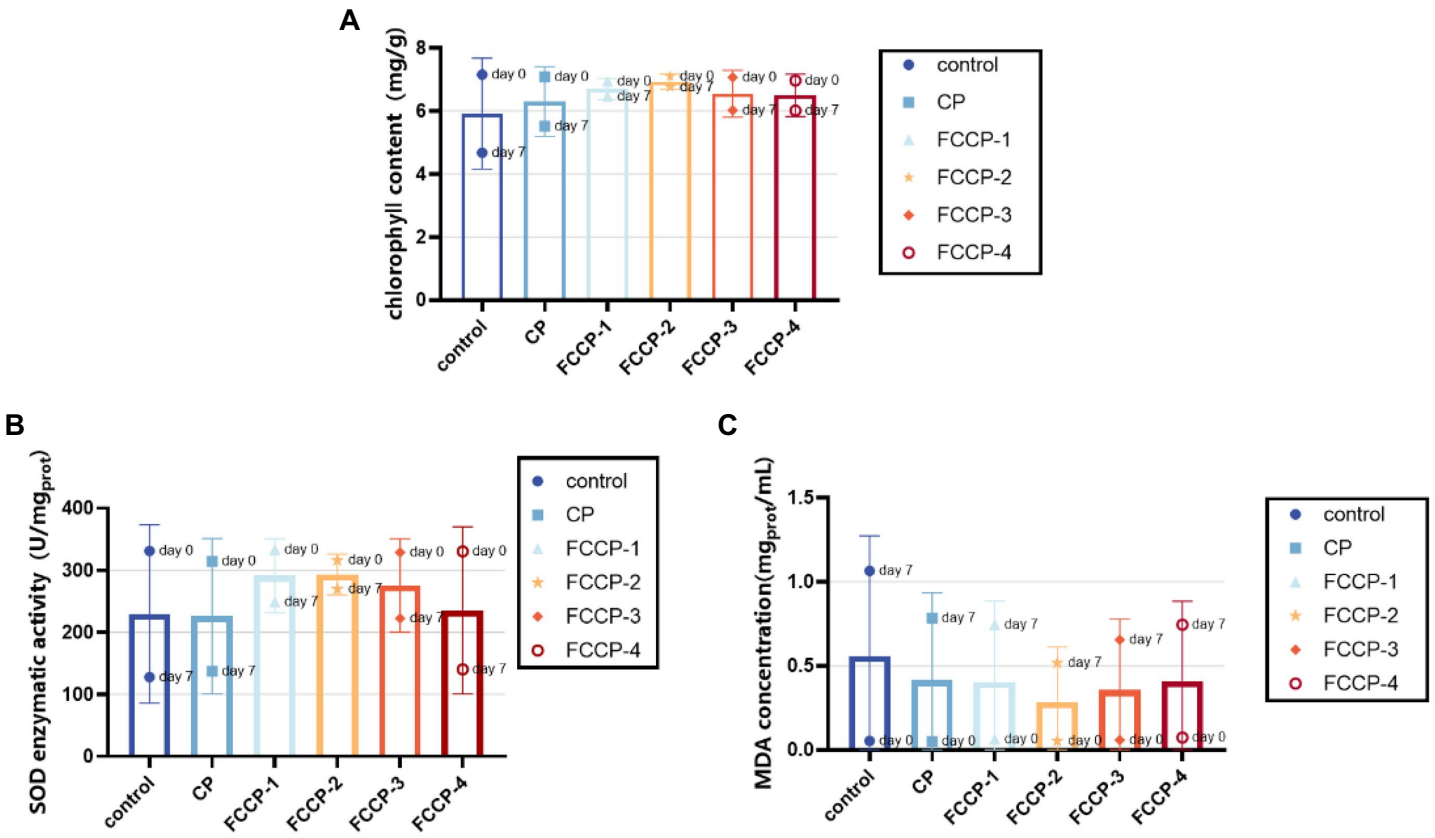
The effect of FCCP on total chlorophyll content **(A)** SOD activity **(B)** and MDA concentration **(C)**.

Chlorophyll degradation occurs in green vegetables during storage, so changes in chlorophyll content are often used as an important indicator of changes in the quality of green vegetables quality (Peng et al., 2015). As shown in Figure 9A, the chlorophyll content of each group was similar on day 0, but the chlorophyll content of fresh-cut lettuce treated with FCCP was consistently higher than that of the control group after 7 days of storage, indicating that FCCP can inhibit chlorophyll loss from fresh-cut lettuce, with the minimal change chlorophyll content of 6.78 mg/100 g after FCCP-2 treatment, and after 7 days of storage at 4°C, the fresh-cut lettuce of the control and CP groups The chlorophyll content decreased to 4.67 and 5.52 mg/100 g. The differences in chlorophyll content between treatment and control groups and between treatment groups were significant (*p* > 0.05) and consistent with the results of the above chromaticity study.

### 3.12. Analysis of SOD activity and MDA concentration

As shown in Figure 9B, the content of superoxide dismutase (SOD) determines the rate of oxygen radical production by fruit and vegetable cells during storage. Higher SOD activity indicates that fresh-cut lettuces can maintain its reactive oxygen metabolism balance. At the beginning of storage, mechanical damage resulted in higher SOD enzyme activity, but as fresh-cut lettuce was stored at 4°C for 7 days, SOD activity was significantly higher in the FCCP-2 treated group than in the control group (*p* < 0.05). The SOD activities of the control, CP and FCCP-2 groups were 127.8 U/mgprot, respectively. 137.4 U/mgprot and 269.9 U/mgprot, which indicated that FCCP has an important role in maintaining the activity of reactive oxygen scavenging enzymes in fresh-cut lettuce, preventing the accumulation of reactive oxygen species and the occurrence of membrane lipid peroxidation, delaying senescence and inhibiting fruit softening.

Malondialdehyde content is a key index to evaluate the degree of aging and membrane peroxidation of fruits and vegetables. Figure 9C shows that the MDA content was low at day 0. The control group reached 1.06 mgprot/mL after 7 days of storage at 4°C, and its MDA content was 51. 48% higher than that of the FCCP-2 treatment group, indicating that the malondialdehyde content of the FCCP treatment group and the control group after 7 days of storage of fresh-cut lettuce. The significant difference (*p* < 0.05) in the content of malondialdehyde between the FCCP-treated and control groups after 7 days of storage indicates that the FCCP-coated treatment was better able to limit the membrane peroxidation process, thus maintaining the integrity of lettuce cells. Overall, it indicates that FCCP inhibits browning of fresh-cut lettuce by inducing SOD activity, reducing MDA accumulation, and mitigating lipid peroxidation.

## 4. Discussion

Organic solvent extraction, enzyme-assisted extraction, ultrasonic extraction, microwave extraction, and recently developed extraction and separation techniques including supercritical fluid extraction, subcritical water extraction, and ionic liquid extraction can all be used to extract FSL (Liu et al., 2022). Since it was found in this investigation to have a higher extraction rate and be more suitable for large-scale production, the ultrasonic-assisted extraction method was chosen for extraction. The purification of FSL-1 was found to work optimum with polyamide resin (Zhang et al., 2010) and AB-8 macroporous resin (Cui et al., 2018). In order to increase the race of FSL-1 extraction and purification, this research combined the practical use of these two purification techniques for the first time, and the purification rate was increased by 18.53%. Chitosan preservation products can frequently be used to store fresh-cut fruits and vegetables. Infrared spectroscopy has shown that the presence of many hydroxyl groups in phenolics can interact with the hydroxyl groups in chitosan to create hydrogen groups, improving FCCP capacity to form films. One of the most important elements in the success of the surface coating of fresh-cut fruits and vegetables is the coating solution’s viscosity. The addition of FSL-1 affected the chitosan matrix, which in turn increased the preservation solution’s viscosity, resulting in a more uniform formation of the surface coating by FCCP (Zhang et al., 2010). By measuring the contact angle of FCCP on glass and polystyrene plastic plates, it can be seen that the nature of chitosan film is significantly influenced by FSL-1 and concentration (Wang et al., 2011; Jagodzinska et al., 2021). Fresh-cut fruits and vegetables have both a high viscosity adhesion and a certain wettability to diffuse on their surface. With increasing FSL-1 and concentration, the film-forming solution’s wettability on the glass plate dramatically decreased while just marginally declining on the polystyrene substrate. As a result, FCCP has higher film-forming and water resistance qualities.

The application of FSL-1 in naturally occurring compound food preservatives has not been studied yet, despite flavonoid extracts being often used in natural food preservatives (Jiang et al., 2022). Fresh-cut fruits and vegetables are very susceptible to microbial infestation such as foodborne pathogens during preservation leading to rapid decay and shortened shelf life, so natural food preservatives with excellent antibacterial ability make very necessary, and according to available reports, FSL-1 has the strongest antibacterial activity against *S. aureus* (Criste et al., 2020), consistent with the results of this experimental study. The antimicrobial activity of FCCP was mainly derived from CS. The antimicrobial mechanism of CS is based on electrostatic interactions between protonated amino groups and negatively charged residues on the surface of microorganisms, leading to leakage of intracellular components that exert antimicrobial activity (Hosseinnejad and Jafari, 2016). The addition of flavonoids significantly enhanced the antimicrobial activity of the preservative. Researchers have proposed several mechanisms to explain the antimicrobial activity of flavonoids: (1) flavonoids can form compound with cell wall components and inhibit microbial adhesion; (2) flavonoids can disrupt the integrity of microbial cell membranes (Farhadi et al., 2019); (3) flavonoids can interfere with the synthesis of nucleic acids in microorganisms (Gorniak et al., 2019). Although FCCP showed strong antibacterial activity against *S. aureus* and *E. coli*, while it was weak against *S. typhimurium* and *L. monocytogenes*. It is worth noting that the addition of flavonoids enhanced the antimicrobial activity of the preservatives to different degrees. Fruits are easily oxidized when exposed to air, and lead to the reduce of skin resistance, which causes the susceptibility to disease by microorganisms, and results in loss of nutrients and a decline in quality. In addition, the infection of external pathogens can cause the oxidative stress reaction inside the fruit to establish the defense system, which also accelerates the senescence of the fruit (Wang et al., 2019). The packaging materials with optimal antioxidant properties can block the invasion of free radicals and act as a barrier on the fruit surface. Therefore, it is necessary to test the antioxidant capacity of the coating materials, and the addition of natural substances such as flavonoids can improve the antioxidant capacity, and the results of this study showed that the results are generally consistent with previous studies (Shaheen et al., 2016), and FSL-1 and FCCP materials have outstanding scavenging effects on DPPH, ABTS, and total antioxidant capacity, but the chitosan preservative alone antioxidant capacity was weak, and the results showed that FSL-1 and CS made into a composite preservative could greatly improve the antioxidant capacity of CP. In other words, the antibacterial ability of FSL-1 and the antioxidant ability of CP can be perfectly combined to obtain a coating material with both antibacterial and antioxidant abilities.

Fresh-cut lettuce’s appearance was shown to be substantially affected by browning and dehydration after receiving treatment in the blank group. However, those treated with FCCP coating kept their appearance intact, and then the quality evaluation of fresh-cut lettuce was derived from some indicators. Firstly, the weight loss of the control and experimental groups stored at 4°C for 7 days was compared, and it was found that the weight loss rate of the experimental group was significantly lower than that of the control group, which indicated that the FCCP coating treatment could effectively reduce the weight loss of fresh-cut lettuce, probably due to the fact that FCCP affected the permeability of lettuce to CO_2_, O_2_ and water vapor, reducing the evaporation of water through the pores of the lettuce epidermis, thus allowing the internal pressure to saturate and thus reducing the weight loss rate (Nor and Ding, 2020). Hardness is also an important index to judge fruit maturity and quality evaluation, for the increased activities of the enzyme related to the cell degradation during storage, which is susceptible to bring the loss of firmness, even the microbial invasion. The L* and b* values of fresh-cut lettuce gradually decreased during storage, while the a* values gradually increased, but chlorophyll degradation occurs in green vegetables during storage, so the chlorophyll content was consistent with the results of the study about BI. It was found that chlorophyll content, SOD activity and MDA content were higher after FCCP treatment than in the control group, suggesting that FCCP can protect chlorophyll loss, maintain SOD enzyme activity and protect cells from damage. The structure of the study is consistent with other reports (Li et al., 2021a) It is basically consistent that FCCP helps to maintain the quality of fresh-cut lettuce and enhance the nutritional value of fresh-cut lettuce. Importantly, in the experimental group of FCCPs, FCCP-2 showed the best freshness preservation performance in fresh-cut lettuce with the lowest weight loss of only 15.23%, the smallest variation in hardness and BI, the highest SOD enzyme activity and the lowest MDA content, indicating that the optimal use of FSL-1 in fresh-cut lettuce applications should be 1 mg/ml, which is in accordance with the permissible addition of natural preservatives to food. In conclusion, FCCP can be used as a natural food preservative to extend the shelf life of fresh-cut fruits and vegetables. It provides research basis and direction for the development and utilization of SBL in the pharmaceutical, food and health product industries, so that SBL can be turned into treasure to a greater extent and the added value of sea buckthorn resources can be improved.

However, there are still shortcomings in this study, the inhibition of fungi (*Aspergillus niger* and *Penicillium*) by FSL was not evident in the preliminary experiments and the ability of this preservative against fungi was not analyzed. In addition, monthly variations may affect the concentration of antioxidant active compounds in sea buckthorn, and the side effects on humans regarding the dose used or the high dose have not been studied. Subsequent experiments will investigate more specifically the composition and content of the experimental sea buckthorn leaf flavonoids, using HPLC or LC–MS instruments for the quantification and characterization of the purified extracts. In recent years, with the in-depth research and application development of sea buckthorn leaves, related products have been released. Therefore, there is a wide prospect of developing new food, health food and pharmaceuticals from sea buckthorn leaves.

## 5. Conclusion

The combination of chitosan and FSL-1 creates a new type of complex preservative. The antibacterial and antioxidant properties of FCCP were greatly enhanced by the addition of FSL-1. Fourier transform infrared spectroscopy data showed that FSL interacted with chitosan to produce intermolecular hydrogen bonds, which allowed FSL-1 to bind to the CS matrix. The results showed that the viscosity and contact angle of the composite preservative increased, with the highest viscosity of 6.88 mPas for FCCP-2 and the maximum contact angle of 31.93° in the glass plate with high hydrophobicity, indicating its superior film-forming properties. The antibacterial properties of the composite film were improved by the addition of FSL-1, among which FCCP-2 had the best antibacterial effect on *S. aureus* (20.74 ± 0.11 mm), but the antibacterial effect on *L. monocytogenes* (14.18 ± 0.27 mm) was poor. The film was coated with FCCP as an active packaging material to preserve fresh-cut lettuce when stored at 4°C for 7 days. Compared with fresh-cut lettuce without film coating, FCCP-2 coated lettuce was superior to the other test groups in terms of weight loss, hardness, browning index, SOD activity and MDA content. Since FSL-1 is made from agricultural waste and has the qualities of resource conservation and sustainability, FCCP has great potential as a food preservation material. Our results showed that FCCPs, especially FCCP-2, added with 1.0 mg/ml FSL-1, showed the best freshness preservation effect on fresh-cut lettuce, indicating that FCCP is a natural food preservative that can extend the shelf life of food by preventing oxidation and deterioration.

## Data availability statement

The original contributions presented in the study are included in the article/supplementary material, further inquiries can be directed to the corresponding author.

## Author contributions

KF, CL, and WH: formal analysis. KF, WT, WZ, SL, and WH: funding acquisition. KF, XF, CL, SL, and WH: investigation. QZ, WT, XF, KF, YL, and WH: methodology. KF, WT, XF, WZ, and YL: project administration. QZ and WT: resources. KF: software and writing—original draft. KF and WH: supervision. All authors contributed to the article and approved the submitted version.

## Conflict of interest

The authors declare that the research was conducted in the absence of any commercial or financial relationships that could be construed as a potential conflict of interest.

## Publisher’s note

All claims expressed in this article are solely those of the authors and do not necessarily represent those of their affiliated organizations, or those of the publisher, the editors and the reviewers. Any product that may be evaluated in this article, or claim that may be made by its manufacturer, is not guaranteed or endorsed by the publisher.
